# Exogenous Kinetin Modulates ROS Homeostasis to Affect Heat Tolerance in Rice Seedlings

**DOI:** 10.3390/ijms24076252

**Published:** 2023-03-26

**Authors:** Wentao Mei, Wenjuan Chen, Yingfeng Wang, Zeyun Liu, Yating Dong, Guilian Zhang, Huabing Deng, Xiong Liu, Xuedan Lu, Feng Wang, Guihua Chen, Wenbang Tang, Yunhua Xiao

**Affiliations:** 1 Hunan Provincial Key Laboratory of Rice and Rapeseed Breeding for Disease Resistance, College of Agronomy, Hunan Agricultural University, Changsha 410128, China; 2 State Key Laboratory of Hybrid Rice, Hunan Hybrid Rice Research Center, Changsha 410125, China

**Keywords:** rice (*Oryza sativa* L.), seedling, cytokinin, heat stress, kinetin

## Abstract

Heat stress caused by rapidly changing climate warming has become a serious threat to crop growth worldwide. Exogenous cytokinin (CK) kinetin (KT) has been shown to have positive effects in improving salt and drought tolerance in plants. However, the mechanism of KT in heat tolerance in rice is poorly understood. Here, we found that exogenously adequate application of KT improved the heat stress tolerance of rice seedlings, with the best effect observed when the application concentration was 10^−9^ M. In addition, exogenous application of 10^−9^ M KT promoted the expression of CK-responsive *OsRR* genes, reduced membrane damage and reactive oxygen species (ROS) accumulation in rice, and increased the activity of antioxidant enzymes. Meanwhile, exogenous 10^−9^ M KT treatment significantly enhanced the expression of antioxidant enzymes, heat activation, and defense-related genes. In conclusion, exogenous KT treatment regulates heat tolerance in rice seedlings by modulating the dynamic balance of ROS in plants under heat stress.

## 1. Introduction

With the increasing population and the spread of industrialization in recent years, global warming has become an imminent problem [1]. The global warming trend is faster, longer, and more damaging than we expected [2]. Reports show that, from 1880 to 2012, the global average temperature rose by 0.85 °C. By 2100, the global average surface temperature in Southeast Asia is expected to rise by 3–5 °C, with many parts of the tropics reaching their highest temperatures on record in the future [3]. Heat stress due to global warming severely constrains crop yields [4]. According to statistics, without reasonable fertilizer ratios, natural variation, and genetic breeding improvements, the yield of major food crops such as wheat, rice, corn, and soybeans will decrease by 6.0%, 3.2%, 7.4%, and 3.1%, respectively, for every 1 °C increase in global average temperature [5]. However, as the population grows, crop yields must increase to sustain the needs of daily life. It is estimated that, to meet the needs of the projected 9 billion people in 2050, food production should ideally increase by 70% [6].

Rice is one of the most important food crops and is also the model plant for monocotyledon research, with nearly half of the world’s population relying on it as a staple food; furthermore, in Southeast Asia, rice provides 70% of the caloric intake [7]. Temperature is one of the important climatic factors affecting the growth of rice. An average daily temperature of 25–30 °C is currently found to be the optimum temperature for rice growth, and heat stress can lead to injury to the floral apparatus, root growth, and seed filling of rice, which directly affects yield and quality [8]. During the vegetative stage, plants treated with heat showed various morphological symptoms, such as wilting, curling and yellowing of leaves, and reduction in tiller number and biomass [9]. In the reproductive stage, heat stress during flowering can lead to other shape changes as well as reduced anther dehiscence [10] and reduced pollen viability [11], which severely impair the process of pollination and fertilization, ultimately leading to reduced fertility. Based on the significance of rice among the world’s food crops and the adverse effects of global warming on rice growth and development, the imminent question is how to improve the tolerance to heat stress at each stage of rice production.

CK has important roles in plant growth and development, including cell division and differentiation [12], apical dominance [13], and leaf senescence [14], among others. In plants, several natural forms of CK exist, including *N*^6^-(Δ^2^-isopentenyl) adenine (iP), *trans*-zeatin (tZ), *cis*-zeatin (cZ), dihydrozeatin, and topolins [15]. Early studies have noted the function of CK in enhancing external stresses based on changes in endogenous CK concentrations in response to stress [16]. In recent years, with the discovery of the *Arabidopsis* CK metabolism and signaling system and the widespread use of CK mutants, the role of CK in various abiotic and biotic stress responses has been widely reported. In CK-deficient plants, levels of D1, the main target protein of photodamage, are strongly reduced, which results in reduced photoprotection and lower recovery from photoinhibition [17,18]. When *Arabidopsis* and soybean respond to external drought and salt stress, the endogenous CK content is reduced through downregulation of the CK synthesis gene *IPT* and upregulation of the CK oxidation gene *CKX*, ultimately reducing plant stress tolerance [19,20]. In contrast, transcriptomic and proteomic studies revealed additional links between CK and the heat response. In *Arabidopsis*, transcriptome changes in response to CK and high temperature overlapped considerably; the proteome response to CK treatment at a standard temperature partially mimicked the effect of heat shock on the proteome [21]. During the reproductive stage of rice, however, heat stress significantly reduces the CK content in the spikes of heat-sensitive rice varieties, and the application of exogenous CK can mitigate heat damage [22]. In conclusion, the available evidence suggests that CK is involved in plant heat stress mechanisms, but the exact function remains unclear.

Kinetin, the first and best known CK, was first isolated from autoclaved herring sperm and has great potential in application areas owing to its favorable synthetic properties; it is now commonly used as an exogenous plant fortifier whose exogenous application can promote plant cell proliferation [23]. Compared with other species of CK, exogenous application of KT has better performance in improving plant stress resistance [24]. Studies have shown that exogenous application of KT can significantly attenuate the effects of radiation on growth, photosynthesis, and nitrogen metabolism in tomato [25]. When maize was exposed to boron toxicity, application of KT enhanced its oxidative defense system and promoted growth [26]. KT interacts with a variety of plant hormones to improve salt tolerance in soybean [27]. Unfortunately, the role of KT in plant heat stress responses remains to be investigated. Here, we investigated the potential mechanisms by which exogenously applied KT affects heat tolerance in rice under heat stress. The results of this study will enhance our understanding of the relationship between CK and plant heat tolerance.

## 2. Results

### 2.1. Exogenous Application of 10^−9^ M KT Significantly Increased Seedling Survival under Heat Stress

**Figure 1:**
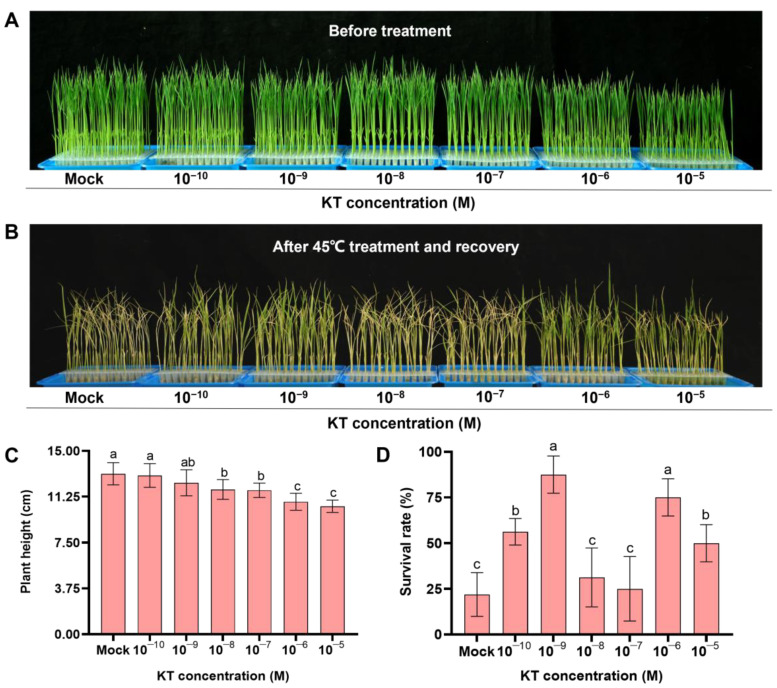
Effect of exogenous application of different concentrations of KT on the survival rate of rice seedlings under heat stress. (**A**) Effect of different concentrations of KT treatment on the growth of ZH11 seedlings at a normal temperature of 28 °C. (**B**) The phenotypes of the different concentrations of KT treatments after high-temperature treatment at 45 °C for 40 h followed by normal temperature recovery at 28 °C for 7 d. (**C**,**D**) Statistical data of plant height (**C**) and survival rates (**D**) in (**A**,**B**), respectively. Data are means ± SD. Different lowercase letters show significant differences among treatments analyzed by one-way ANOVA comparison test (n = 8, *p* < 0.05).

To investigate the effect of applying exogenous CK on heat stress, we treated 4-day-old ZH11 at seedlings with six concentration gradients (10^−10^, 10^−9^, 10^−8^, 10^−7^, 10^−6^, and 10^−5^ M) of exogenous KT for 4 d. The seedlings’ plant height did not differ significantly from the control at a low concentration of KT (10^−10^ and 10^−9^ M) treatment, while it was significantly inhibited as the treatment concentration increased (Figure 1A,C). Subsequently, 8-day-old seedlings were transferred to high-temperature treatment at 45 °C for 40 h, followed by recovery at a normal temperature of 28 °C in solution without KT for 7 d. It can be seen that exogenous application of low concentrations (10^−10^ and 10^−9^ M) and high concentrations (10^−6^ and 10^−5^ M) of KT significantly enhanced seedling heat tolerance, and that the seedlings treated with 10^−9^ M KT had the highest survival rate (Figure 1B,D). The results suggested that 10^−9^ M KT has the most significantly positive effect on heat tolerance, but a relatively small effect on the growth of rice seedlings.

### 2.2. Exogenous KT Attenuates the Effect of Heat Stress on Membrane Lipid Peroxidation in Seedlings

Relative electrolyte leakage rate, antioxidant enzyme activity, and malondialdehyde (MDA) content are often used as indicators of membrane and oxidative damage and reflect the heat tolerance of plants [5]. To further reveal the potential physiological mechanism of exogenous KT treatment to improve heat tolerance in rice, 10^−9^ M KT, which had the most significant heat tolerance effect, was selected as the follow-up experimental concentration, and the malondialdehyde content (MDA), relative electrolyte leakage rate, superoxide dismutase (SOD) activity, and peroxidase (POD) activity of 10^−9^ M KT and control plants under high-temperature stress were measured.

**Figure 2:**
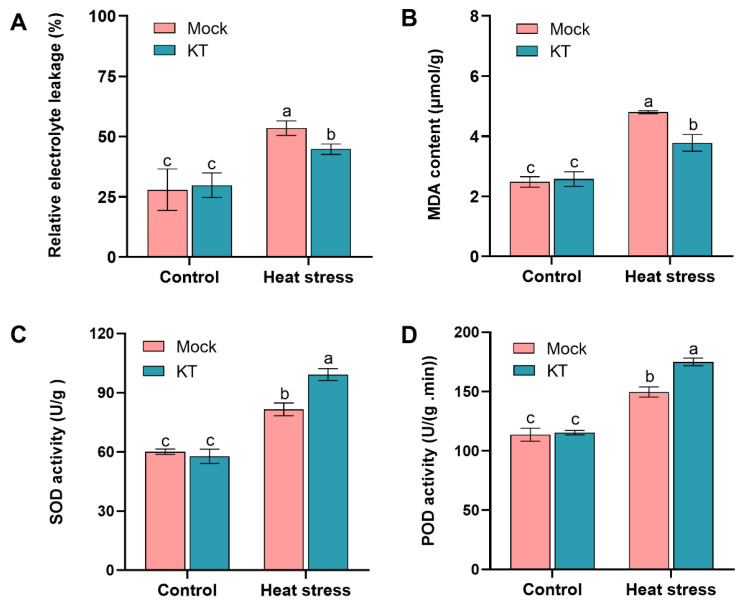
Effect of exogenous KT treatment on membrane lipid peroxidation and antioxidant enzyme activities in rice seedlings under heat stress. Relative electrolyte leakage rate (**A**), malondialdehyde content (**B**), superoxide dismutase activity (**C**), and peroxidase activity (**D**) of 10^−9^ M KT and mock groups after high-temperature treatment at 45 °C. Data are means ± SD. Different lowercase letters show significant differences among treatments analyzed by one-way ANOVA comparison test (n = 3, *p* < 0.05).

There was no significant difference in MDA content and relative conductivity between KT and control seedlings under control conditions at moderate temperatures (Figure 2A,B). After 40 h of heat stress at 45 °C, the MDA content of both KT and control seedlings was significantly increased, but the increase in the MDA content and relative conductivity of KT was significantly lower than that of the control (Figure 2A,B). The results showed that exogenous application of KT reduced the membrane damage suffered by plants under heat stress.

We also examined the SOD and POD activities of KT and control seedlings under heat stress. The data showed that both antioxidant enzyme activities of KT and control seedlings were higher than the control after 40 h of heat stress, and the increase in KT was significantly higher than that of the control (Figure 2C,D). These results suggest that exogenous application of KT under heat stress can increase the antioxidant enzyme activity of seedlings.

### 2.3. Exogenous KT Attenuates the Effect of Heat Stress on Reactive Oxygen Species (ROS) Levels in Seedlings

**Figure 3:**
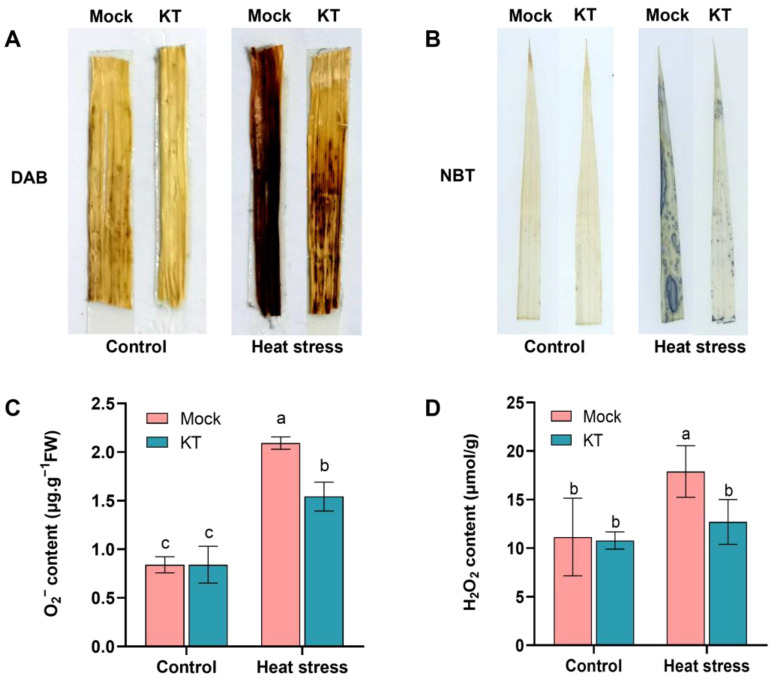
Effect of exogenous KT treatment on ROS levels in seedlings under heat stress. NBT staining (**A**), DAB staining (**B**), O_2_^−^ content (**C**), and H_2_O_2_ content (**D**) test of 10^−9^ M KT versus mock seedlings under heat stress. Data are means ± SD. Different lowercase letters show significant differences among treatments analyzed by one-way ANOVA comparison test (n = 3, *p* < 0.05).

Reactive oxygen species (ROS) levels are also commonly used as a basis for evaluating the heat tolerance of the plant. It was shown that the level of intracellular ROS was significantly increased under high-temperature conditions [28]. To investigate whether exogenous KT treatment could enhance ROS resistance, we examined the accumulation of H_2_O_2_ and O_2_^−^ under heat stress. The 8-day-old KT and control seedlings were treated at 45 °C for 40 h, followed immediately by DAB and NBT staining, and it was found that the leaves of KT and control seedlings did not differ significantly under control conditions, whereas the leaves of KT seedlings were stained lighter than the control under heat stress (Figure 3A,B). The H_2_O_2_ and O_2_^−^ contents of the plants were further measured and, in agreement with the staining results, the amounts of H_2_O_2_ and O_2_^−^ accumulated in the control group under heat stress were significantly higher than in the KT group, with the H_2_O_2_ content being about 1.5 times higher and the O_2_^−^ content about 1.4 times higher than in the KT group (Figure 3C,D). It was evident that the KT group accumulated less ROS compared with the control group. The results showed that exogenous application of KT under heat stress could reduce oxidative damage in the plants.

### 2.4. Exogenous KT Enhances CK Response and Antioxidant-Related Genes’ Expression in Seedlings under Heat Stress

**Figure 4:**
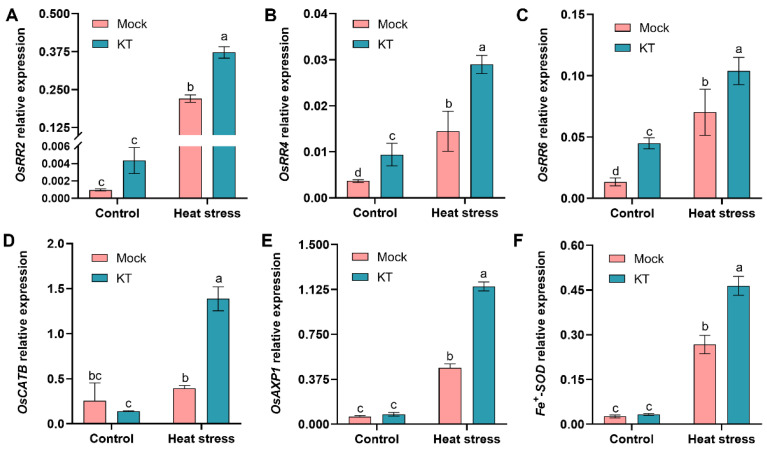
Relative expression of CK response and antioxidant-related genes in 10^−9^ M KT under heat stress. For qRT-PCR, *OsActin1* was used as internal reference to analyze the relative expression of CK response genes, *OsRR2* (**A**), *OsRR4* (**B**), and *OsRR6* (**C**), and antioxidant-related genes, *OsCATB* (**D**), *OsAXP1* (**E**), and *Fe^+^-SOD* (**F**). Data are means ± SD. Different lowercase letters show significant differences among treatments analyzed by one-way ANOVA comparison test (n = 3, *p* < 0.05).

To elucidate the possible molecular mechanism of exogenous KT to enhance heat tolerance, the transcript levels of six genes related to CK response and antioxidant were examined in KT and control plants under control and heat stress conditions using RT-qPCR. Among them are three antioxidant-enzyme-related genes (peroxidase-encoding gene *OsCATB*, superoxide dismutase encoding gene *Fe^+^-SOD*, and ascorbate peroxidase *OsAXP1*) [29,30,31] and three A-type *OsRR* genes (*OsRR2*, *OsRR4*, and *OsRR6*). The A-type *OsRR* gene is a gene typically induced by CK expression and is usually used as a marker gene to analyze the CK response within the plant, thus reflecting the activity of CK signaling [32,33,34]. Firstly, we evaluated the transcriptional changes of CK response genes between KT and control plants under heat stress. As shown in the figure, the transcript levels of all three CK-responsive genes exhibited a greater degree of upregulation under heat stress in KT plants than in the control (Figure 4A–C). The transcript levels of *OsRR2*, *OsRR4*, and *OsRR6* were also higher in KT plants than in the control under ambient conditions, also indicating that exogenous KT treatment led to an increase in endogenous CK content in the plants (Figure 4A–C). Thus, exogenous KT treatment positively activated the expression of CK-responsive genes under heat stress. Unlike CK-responsive genes, there was no significant difference between the antioxidant enzyme genes of KT and control seedlings under ambient conditions. However, after heat stress, the expression of antioxidant enzyme genes was upregulated in both KT and control seedlings, and the degree of upregulation was significantly higher in the KT group than in the control group (Figure 4D–F). The results of transcript levels can also indicate that the KT antioxidant enzyme activity was higher than that of the control, which is consistent with the results of the antioxidant enzyme activity assay described above. The above results indicate that KT drives the expression of CK response and antioxidant-related genes in rice under heat stress.

### 2.5. Exogenous KT Enhances Heat-Tolerance- and Defense-Related Genes’ Expression in Seedlings under Heat Stress

**Figure 5:**
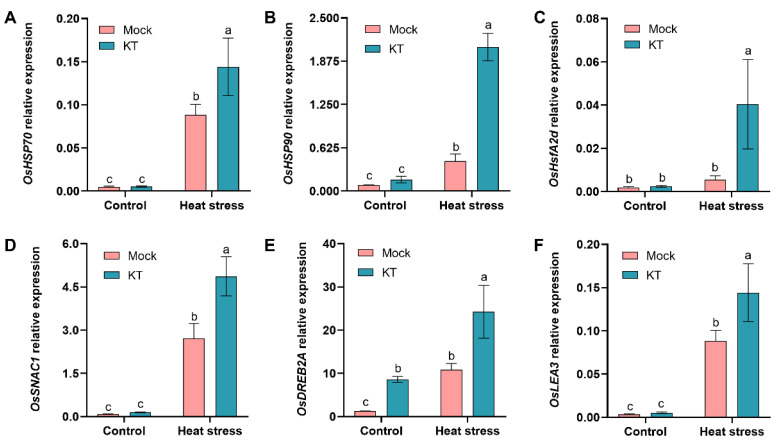
Relative expression of heat-tolerance and heat-defense-related genes in 10^−9^ M KT under heat stress. For qRT-PCR, *OsActin1* was used as an internal reference to analyze the relative expression of heat tolerance genes, *OsHSP70* (**A**), *OsHSP90* (**B**), and *OsHsfA2d* (**C**), and heat-defense-related genes, *OsSNAC1* (**D**), *OsDREB2A* (**E**), and *OsLEA3* (**F**). Data are means ± SD. Different lowercase letters show significant differences among treatments analyzed by one-way ANOVA comparison test (n = 3, *p* < 0.05).

The main function of heat shock proteins (HSPs) is to regulate protein folding and unfolding, and they are typical heat response proteins [5]. Furthermore, the heat shock factor A2 (HsfA2) family has been reported to play an important role in plant heat tolerance, and overexpression of *OsHsfA2d* induced the expression of many *HSP* genes [35]. Therefore, the expression of *OsHSP70*, *OsHSP90*, and *OsHsfA2d* was examined between KT and control seedlings under heat stress using RT-qPCR. The transcript levels of *OsHSP70*, *OsHSP90*, and *OsHsfA2d* were significantly higher in the KT group than in the control group after 40 h of heat stress. In contrast, there was no significant difference in the expression levels of these three heat-tolerance-related genes between KT and control seedlings under ambient conditions (Figure 5A–C).

Three stress defense genes (stress-responsive NAC transcription factor gene *OsSNAC1*, dehydration-responsive element binding protein 2A *OsDREB2A*, and late embryonic enrichment protein gene *OsLEA3*) [4] have been reported to play a key role in the exposure of rice to heat stress. Subsequently, changes in the expression of *OsSNAC1*, *OsDREB2A*, and *OsLEA3* were examined in KT and control groups under heat stress. Under heat stress, the transcript levels of all three defense-related genes were significantly higher in KT than in the control, with *OsSNAC1* being the most significantly upregulated (Figure 5D–F). Notably, the transcript levels of *OsDREB2A* at KT were significantly higher than those of the control under ambient conditions, suggesting that its expression may be induced by exogenous CK (Figure 5E). The above results indicate that KT induces the expression of heat tolerance and heat-defense-related genes in rice under heat stress.

## 3. Discussion

With the intensification of the “greenhouse effect” in recent years, heat damage has become an important factor threatening the yield and quality of rice. Chemical kinetochores that enhance plant defenses can rescue self-regulatory systems damaged by persistent or intense heat and are thus considered promising tools for plant protection and sustainable agriculture [36]. KT was the first CK to be synthesized and has now been reported to improve salt tolerance and promote cell division and differentiation in plants. In this study, we reported the effect of exogenous application of KT on tolerance to high-temperature stress in rice seedlings. Our results suggest that exogenous application of KT can improve heat stress tolerance by regulating antioxidant capacity and transcript levels of endogenous cytokinin marker genes as well as defense- and stress-related genes.

In recent years, the synthetic cytokinin KT has been used as a growth regulator to improve the growth of plants under stress. The studies showed that a proper KT concentration can improve salt tolerance, increase chlorophyll content, delay leaf senescence, and promote plant growth and development [37]. In this study, we found that the survival rate of seedlings in the experimental group was significantly higher than that of the control group after 40 h of high-temperature treatment at 45 °C by exogenous application of 10^−10^, 10^−9^, 10^−8^, 10^−7^, 10^−6^, and 10^−5^ M KT to ZH11 seedlings (Figure 1A,B,D), indicating that exogenously adequate application of KT improved the heat stress tolerance of rice seedlings. High cytokinin treatment inhibits the growth of aboveground and root systems of rice seedlings [38]. We found that, in the experimental group, with the increase in exogenous KT concentration, the above-ground plant height of rice seedlings was significantly reduced and growth was significantly inhibited (Figure 1C). Based on the above results, we finally selected 10^−9^ M, which most significantly enhanced the heat resistance of seedlings, as the experimental group to investigate the possible mechanism of exogenous KT to enhance the heat resistance of rice seedlings without affecting the normal growth of seedlings.

Damage to plant growth and development caused by heat stress is usually associated with the disruption of physiological and metabolic processes in plant cells. Biofilms have a highly ordered structure composed of lipids and proteins that serve as the main barrier for plants and are considered to be the most sensitive component of plant cells to high temperatures [39]. Elevated temperatures can destroy the structure and function of cell membranes, triggering protein denaturation, resulting in compromised membrane integrity and increased leakage of organic and inorganic ions from the cell [39,40]; therefore, the accumulation of MDA and the relative electrolyte leakage rate can well reflect the degree of cell damage. Under heat stress, the MDA content and relative electrolyte leakage rate of KT were significantly lower than those of the control, indicating that exogenous KT attenuated the membrane damage caused by the high temperature (Figure 2A,B).

When plants continue to suffer from heat stress, intracellular ROS will accumulate in excess, causing oxidative damage and eventually leading to cell death and even seedling death. At the same time, the high temperature also impairs the activity of antioxidant enzymes, especially SOD and POD [5], which is consistent with our results (Figure 2C,D). Under continuous heat stress, the activities of SOD and POD increased in both the KT group and the control group, with the most significant increase in the KT group, corresponding to its lower O_2_^−^ and H_2_O_2_ contents than the control group, thus reducing cell membrane damage and oxidative damage (Figure 2C,D and Figure 3). Therefore, exogenous KT may be used to modulate the normal structure and function of cells under a high temperature by regulating the activity of plant oxidative enzymes and the content of osmoprotective substances to scavenge toxic substances such as free radicals.

Consistent with the results on antioxidant enzyme activity, we detected significant upregulation of transcript levels of antioxidant-enzyme-related genes *OsCATB*, *Fe^+^-SOD*, and *OsAXP1* under heat stress (Figure 4D–F). This suggests that exogenous KT may enhance the activity of antioxidant enzymes and scavenge ROS under heat stress by activating the expression of antioxidant-enzyme-related genes, thereby improving high-temperature tolerance in rice. Similar to the results in this paper, it was reported that heat stress upregulated the respiratory burst oxidase (RBOH) homolog of the gene, leading to an increase in ROS content in rice [41]. This conclusion is corroborated by the fact that overexpression of *OsANN1* enhances heat tolerance by promoting CAT and SOD activities [42].

In addition, the endogenous hormone content of plants is also closely related to oxidative stress mechanisms. Recent studies have shown that *OsNCED1* reduces membrane damage and ROS levels in plants under heat stress by regulating ABA content [43]. Therefore, we also examined the transcript levels of CK-responsive genes in the KT and control groups under heat stress; these genes can be used as markers to evaluate the cytokinin response. The transcript levels of *OsRR2*, *OsRR4*, and *OsRR6* were significantly higher in the KT group than in the control group, indicating that the CK response is more active in the KT group under heat stress (Figure 4A–C). Previous studies have found that heat stress promotes *CaIPT5* expression in pepper, leads to an elevated endogenous tZ content in the plants, and enhances heat tolerance [44]. This is consistent with our results, in which *OsRR* expression was significantly higher after heat stress in the control group, indicating that plants enhance the in vivo CK response to withstand external high temperatures (Figure 4A–C). Thus, we speculate that exogenous KT treatment may enhance the heat tolerance of rice by enhancing CK response activity. Increased endogenous CK concentration in tobacco and bryophytes leads to increased antioxidant system activity and upregulation of heat shock protein expression [45,46]. Consistent with this result, the transcript levels of heat response genes *OsHSP70*, *OsHSP90*, and *OsHsfA2d* were significantly upregulated in the KT group compared with the control group under heat stress, consistent with the trend of increasing CK concentration (Figure 5A–C). CK treatment was reported to increase the heat tolerance of maize reproductive tissues and initiate heat stress defense mechanisms [47]. We also found that the transcript levels of heat-defense-related genes *OsSNAC1*, *OsDREB2A*, and *OsLEA3* were significantly upregulated in the KT group compared with the control group under heat stress (Figure 5D–F), indicating that exogenous KT treatment initiated the heat defense mechanism and improved seedling heat tolerance in rice under heat stress. In conclusion, exogenous KT treatment enhanced the antioxidant capacity and thermal defense of rice seedlings by promoting CK response activity, thereby effectively modulating the dynamic balance of plant ROS and reducing membrane damage under heat stress. However, the specific mechanism of exogenous KT treatment to modulate the dynamic balance of ROS under heat stress needs further study.

## 4. Materials and Methods

### 4.1. Plant Materials and Growth Conditions

The *japonica* cultivar Zhonghua11 (ZH11) was used in this study. ZH11 seedlings plants were grown hydroponically in a growth chamber at 28 °C or 45 °C, a 12 h day/12 h night cycle, light intensity of 540 µmol/m^2^ · s, and humidity of 70%. Modified Kimura B (pH 5.8) solution was supplied as the nutrient medium [48]. All plant materials were from Hunan Agricultural University.

### 4.2. Hormone and Heat Stress Treatment

Four-day-old ZH11 seedlings were treated with 10^−10^, 10^−9^, 10^−8^, 10^−7^, 10^−6^, and 10^−5^ M KT for 4 d, and then moved to high-temperature treatment at 45 °C for 40 h followed by recovery at a normal temperature of 28 °C in solution without KT for 7 d. After that, the phenotypes of the treated and control plants were photographed and analyzed.

### 4.3. Measurement of Physiological Indexes

The 10^−9^ M KT experimental and control rice seedlings were treated in a growth chamber after 8 days of growth and exposed to heat stress (45 °C) for 40 h, while those in a growth chamber at 28 °C served as controls. After 40 h of treatment, plants under heat stress and control conditions were used for the determination of relative electrolyte leakage rate, malondialdehyde (MDA) content, hydrogen peroxide (H_2_O_2_) content and superoxide anion (O_2_^−^) content, superoxide dismutase (SOD) activity, and peroxidase (POD) activity, and three biological replicates were performed. The relative electrolyte leakage rate of heat-treated and control plants was measured using the conductivity meter (DS-11A), the MDA content was measured using the thiobarbituric acid (TBA) colorimetric method, the SOD activity was measured using the riboflavin nitro blue tetrazolium (NBT) method, and the POD activity was measured using the guaiacol method [49]. One unit of SOD activity was defined as the amount of enzyme per enzyme extract sample that caused 50% inhibition of the reduction in NBT. The POD activity was calculated with the change in OD_470_ per minute of 0.01 as a relative enzyme activity unit. Referring to the method [50], 3,3-diaminobenzidine (DAB) and nitro blue tetrazolium were used to detect the accumulation of H_2_O_2_ and O_2_^−^ in the leaves of plants. In brief, the H_2_O_2_ content was measured by reacting the extracting solution with 15% NH_4_OH and 10% TiCl_4_ and then measuring the absorbance at 410 nm. The extracting solution of O_2_^−^ was reacted with p-aminobenzenesulfonamide and N-1-naphthylethylenediamin dihydrochloride, and the absorbance of the reaction mixture was determined at 530 nm (BC3595 and BC1295, Solarbio, Beijing, China).

### 4.4. Quantitative Real-Time PCR Analysis

Here, the 10^−9^ M KT and control group plants were heat treated for 40 h and both groups were collected, then the corresponding RNA was extracted after snap freezing with liquid nitrogen. qRT-PCR was used to determine the expression levels of the transcript levels of genes involved in antioxidant enzymes, CK signaling pathway, and heat response and defense. Total RNA extraction was performed using RNA easy isolation reagent (R701-01, Vazyme, Nanjing, China) and reverse transcribed for qPCR analysis using the HiScript^®^ II Q RT SuperMIX for qRT-PCR (+gDNA wiper) kit (R223-01, Vazyme). Rice *OsActin1* was used as an internal reference gene, and primers for amplification were designed by Primer Premier 6.0. The relative changes in gene expression levels were quantitated based on three biological replicates via the 2^−△△Ct^ method. All primer information is shown in Table S1.

### 4.5. Statistical Analysis

Data are presented as mean ± SD and statistical analysis was performed using DPS (version 7.05). Data were analyzed by one-way ANOVA and considered statistically significant at *p* < 0.05. The data were plotted using GraphPad Prism (version 8.01).

## 5. Conclusions

The use of exogenous reinforcers to improve plant stress tolerance is a promising strategy in agricultural production. In this study, we showed that exogenous 10^−9^ M KT treatment could activate the CK response and significantly increase the survival rate of rice seedlings under heat stress. In addition, KT enhanced the heat tolerance of rice by increasing antioxidant enzyme activity, reducing ROS accumulation, and inducing defense-related gene expression, thereby effectively reducing heat-induced oxidative stress. Overall, the physiological and molecular mechanisms of exogenous KT regulating heat tolerance in rice seedlings provide new ideas for rice production under extreme high temperatures.

## Data Availability

The data presented in this study are available upon request from the corresponding author.

## Supplementary Materials

The following supporting information can be downloaded at https://www.mdpi.com/article/10.3390/ijms24076252/s1.
