# Supplementary material for: Exogenous Kinetin Modulates ROS Homeostasis to Affect Heat Tolerance in Rice Seedlings
Source: Int J Mol Sci. 2023 Mar 26;24(7):6252. doi: 10.3390/ijms24076252 (PMC10093947; doi:10.3390/ijms24076252)
Supplement: Supplementary file 1 [file ijms-24-06252-s001.zip › ijms-2291921-supplementary.pdf]

Supplementary Materials

Table S1. The list of RT-qPCR primer pairs.

Primer name	Sequence (5'–3')
OsActin1-F	GACCTTCAACACCCCTGCTA
OsActin1-R	GAGTCCAACACAATACCTGTGG
OsCATB-F	GTTCGGTTCTCCACAGTCGT
OsCATB-R	CCCTCCATGTGCCTGTAGTT
OsAPX1-F	CCAAGGGTTCTGACCACCTA
OsAPX1-R	CAGTTCGGAGAGCTTGAGGT
Fe^{+}SOD-F	CTTGATGCCCTGGAACCTTA
Fe^{+}SOD-R	GCCAGACCCCAAAAGTGATA
OsLEA3-F	TCACTTCAAATTCGGTGCAA
OsLEA3-R	CACACCCGTCAGAAATCCTC
OsDREB2A-F	GGAATCTCCTCCTTTCATCGTG
OsDREB2A-R	TTCCGCTCCTGACAAACACG
OsSNAC1-F	CATGGTCCCGTTCTGAGGTG
OsSNAC1-R	CACACGTTGCAGCATCGATC
OsHSP70-F	GCCAAGCGTCAAGCAGTGACCAA
OsHSP70-R	GGTCATCAAAGCGCCGCCCTAT
OsHSP90-F	TTTGGGCGAAGGTGACACTGCTA
OsHSP90-R	TGGCAATGGTCCCAAGGTTCTTAAT
OsHsfA2d-F	CAAGAGATGATGCTGGGATTCC
OsHsfA2d-R	CTATTGCTTAGATAACCCAGCT
OsRR2-F	CATGGTGATGAATGCATCC
OsRR2-R	TGCTGCCATTGGACCATCT
OsRR4-F	TGAAGCTGCAACAGCTCA
OsRR4-R	AGTGGAGGACAATCTTGG
OsRR6-F	CTTCTTCTGCTGCTGCCTCAAAC
OsRR6-R	GATTGATGCTCTGGTGCCATTTC
